# Thyroid Hormones and Brain Development: A Focus on the Role of Mitochondria as Regulators of Developmental Time

**DOI:** 10.3390/cells14030150

**Published:** 2025-01-21

**Authors:** Filip Vujovic, Ramin M Farahani

**Affiliations:** 1 IDR/WSLHD Research and Education Network, Sydney, NSW 2145, Australia; 2 School of Medical Sciences, Faculty of Medicine and Health, University of Sydney, Sydney, NSW 2006, Australia

**Keywords:** thyroid hormones, brain development, self-organisation, mitochondria, reactive oxygen species

## Abstract

Thyroid hormones (THs) regulate metabolism in a homeostatic state in an adult organism. During the prenatal period, prior to the establishment of homeostatic mechanisms, THs assume additional functions as key regulators of brain development. Here, we focus on reviewing the role of THs in orchestrating cellular dynamics in a developing brain. The evidence from the reviewed scientific literature suggests that the developmental roles of the hormones are predominantly mediated by non-genomic mitochondrial effects of THs due to attenuation of genomic effects of THs that antagonise non-genomic impacts. We argue that the key function of TH signalling during brain development is to orchestrate the tempo of self-organisation of neural progenitor cells. Further, evidence is provided that major neurodevelopmental consequences of hypothyroidism stem from an altered tempo of cellular self-organisation.

## 1. Introduction

The role of thyroid hormones (THs) in brain development has been extensively reviewed, and readers are referred to scholarly articles covering this topic [1,2,3,4,5,6,7]. The research in this field has typically focused on genomic activities of THs during neurodevelopment [8]. This genome-centric view of TH activity is gradually changing as accumulating evidence suggests that non-genomic effects of the hormones in regulating brain development are as important as the genomic influences [9,10]. A pathological condition that clearly illustrates this notion is brain development in congenital hypothyroidism manifested as cretinism [11]. With an incidence of 1 in 3500 live births [12], congenital hypothyroidism significantly affects brain development, as evidenced by a mean intelligence quotient (IQ) of 76 in affected individuals [13]. Interestingly, while induced hypothyroidism negatively influences cerebellar development in animal models, no such negative effect is observed in mice lacking thyroid receptor-α1 [14]. This observation suggests that impairment of non-genomic effects of THs underpin certain neurodevelopmental consequences of hypothyroidism. A further corollary of the findings of the latter study is that non-genomic effects of THs counterbalance the genomic impacts of the hormone on brain development. Aside from a divergence of outcomes, genomic and non-genomic effects are driven by different forms of THs. While L-triiodothyronine (T3) is responsible for receptor-mediated functions of THs, non-genomic effects are mainly driven by 3,5-diodothyronine (3,5-T2), a by-product of T3 degradation [10]. Given the divergence of mediators and outcomes, an objective of this review is to dissect the role of genomic and non-genomic outcomes communicated by TH signalling in neurodevelopment from a mechanistic perspective.

Focusing on the non-genomic impact of THs, evidence suggests that mitochondria mediate some of the reported effects [10,15,16]. The interactions of THs with mitochondria are typically studied in the context of regulation of metabolism in a homeostatic state of an adult organism. However, just as the notion of the role of mitochondria as primarily contributing to homeostasis by provision of ATP has started to change, so has the understanding of the interactions of THs with mitochondria in an emerging paradigm. The new paradigm concerns the role of mitochondria in orchestrating the developmental landscape during the prenatal period, when most homeostatic mechanisms are absent. The paradigm is an extension of a growing body of research suggesting that mitochondrial dynamics and neuronal differentiation are intimately linked [17,18,19,20,21,22]. Exploration of the mechanism underlying mitochondrial facilitation of neuronal differentiation revealed that the so-called mitochondrial metabolic by-products (e.g., thermal flux [23,24,25] and reactive oxygen species [18,26]) that are typically removed by homeostatic mechanisms propel neuronal differentiation. This is hardly surprising, as most homeostatic mechanisms are either absent or ineffective in early stages of development. For example, a functional circulation to dissipate heat is not established until embryonic day 10 in developing mouse embryos [27], and yet heat is generated at a high rate of 30 nW/cell at a much earlier, two-cell stage of embryogenesis [28]. This brings about a window of opportunity, characterised by the relative absence of homeostatic mechanisms, in which mitochondrial metabolic by-products (i.e., abiotic signals) can exceed a threshold level to drive reprogramming of other signalling pathways [23]. Given the documented role of THs in amplifying mitochondrial generation of these abiotic signals [29,30], a second goal of the review is to explore whether non-genomic effects of THs in early neurodevelopment are mediated by mitochondrial abiotic signals. We propose that THs orchestrate brain development in a bistable manner. Non-genomic effects emerge first and require amplification of mitochondrial metabolic by-products that regulate multicellular self-organisation during brain development. Genomic receptor-mediated effects are delayed compared to non-genomic effects and counterbalance the impact of mitochondrial activities. Finally, we provide evidence that the genomic and non-genomic signalling activities of THs can be transiently uncoupled to enhance the impact of non-genomic effects during brain development. This proposal is validated in the light of existing evidence regarding the impact of altered thyroid activity on brain development.

## 2. An Overview of the Signalling Landscape of Thyroid Hormones

**Figure 1:**
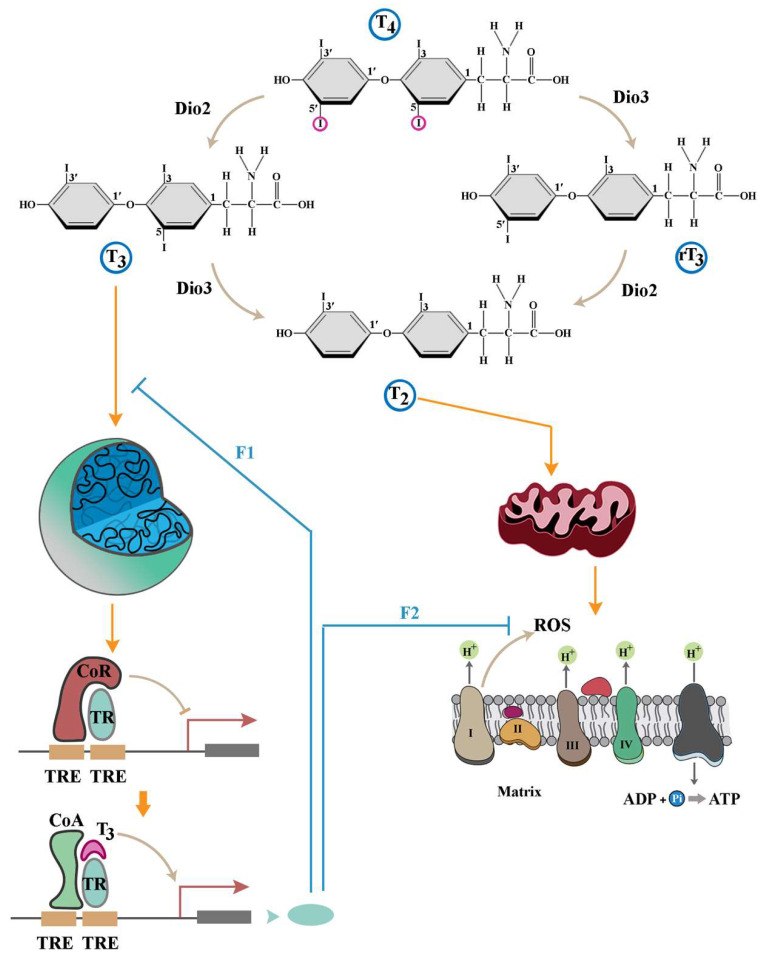
An overview of TH signalling. Genomic effects of THs are triggered by association of T3 with TRs, an event that relieves the inhibitory activity of TR-bound co-repressor complex (CoR) by inducing conformational change in TRs and the resultant recruitment of a co-activator complex (CoA). Genes activated by this mechanism provide negative feedback to T3-mediated signalling (F1) or counteract the non-genomic impact of T2 (F2). Downstream signalling by T2 is mainly mediated by direct reprogramming of the mitochondrial electron transport chain leading to an overproduction of ROS.

A detailed account of the regulation of THs can be found in a recent review by van der Spek et al. (2017) [31], while the overview of TH signalling provided here is a prelude to the discussion that follows. The thyroid gland releases 3,5,3′,5′-tetraiodothyronine (also known as T4 or thyroxine) into the bloodstream. The enzymatic activity of 5′-deiodinase (5′-D, dio2) then converts T4 to T3, the active form that binds to thyroid hormone receptors (TRs). A subsequent deiodination event catalysed by 5-deiodinase (5-D, dio3) converts T4 and T3 to rT3 (reverse T3) and T2, respectively. Although the impacts of different forms of THs are diverse and somewhat context-dependent, the elemental outline of TH signalling is shaped by two general principles. Outcomes induced by THs either depend on transcription- (genomic effects) or occur independently of nuclear dynamics (non-genomic effects) [30] (Figure 1). Further, genomic and non-genomic interactions appear to invoke antagonistic outcomes as detailed below.

Genomic effects of THs are mediated via binding of T3 to different isoforms of nuclear thyroid receptor-α (TRα) and TRβ [30]. The transcriptional outcomes elicited by this complex depend on the target cell. In the pituitary and thyroid glands, the T3/TR complex inhibits expression of the genomic loci encoding the thyrotropin-α and β subunits and thyrotropin-releasing hormone [32], thereby exerting a negative feedback input on production of THs by the thyroid gland. In other peripheral target tissues, the genomic response to THs is context-dependent. The consensus view is that the complex of T3/TR binds to thyroid hormone response elements (TREs) in a homo- or heterodimeric form in the regulatory regions of specific genes to activate transcription of these loci. While the receptor-mediated activity of THs drives a plethora of adaptive changes [32,33], closer examination reveals a generic pattern of regulation of peripheral gene expression to attenuate the consequences of non-genomic functions of THs. An example of the latter activity is observed during T3-mediated upregulation of genes that contribute to glutathione synthesis [34]. Reduced glutathione is the key mammalian non-enzymatic antioxidant [35] essential for the elimination of reactive oxygen species (ROS) of mitochondrial origin that are amplified by non-genomic impacts of THs [36,37]. In the absence of THs, the hormone-free TR recruits a corepressor complex with histone deacetylase activity, which inhibits the latter genomic loci with a binding motif for TRs [32] (Figure 1). Upon exposure to THs, just as T2 amplifies mitochondrial production of ROS, formation of the T3/TR complex reinitiates transcription from these TR-inhibited loci [32], leading to enhanced synthesis of glutathione to eliminate the generated ROS. As expected from the antagonistic interaction of non-genomic and TR-mediated genomic impacts of THs, experimental knockdown of TRs not only does not abolish signalling by THs but also leads to an accelerated progression of developmental events mediated by these hormones [38,39]. Further, compared to the severe consequences of TH deficiency, mice with a deletion of both TRα1 and TRβ exhibit milder phenotypes including a hyperactive pituitary–thyroid axis [40]. A hyperactive pituitary-thyroid axis is consistent with a lack of negative feedback communicated via the T3/TR complex to genomic loci encoding proteins that synthesise THs and thyrotropin. Acceleration of developmental dynamics in TR-null animal models, on the other hand, suggests a dominance of TR-independent non-genomic functions of THs that are otherwise dampened by TR-mediated negative feedback. An interpretation of the accelerated development in TR-null animals is that dynamics of organogenesis are, in part, orchestrated by non-genomic effects of THs.

The non-genomic functions of THs are mediated by interaction of the hormones with mitochondria or other cytoplasmic entities (Figure 1). Non-genomic mitochondria-independent functions of THs have been reviewed in detail by Cheng et al. [30]. A characteristic feature of the non-genomic cytoplasmic effects of THs is the short timeframe of occurrence. A major cluster of non-genomic activities of THs develop within seconds of exposure to the hormones and prime the cell for activation of the citric acid cycle and electron transport chain. T3 induces a rapid increase in cytoplasmic [Ca^2+^] [41] and glucose uptake [42,43]. A subsequent activation of Ca^2+^-ATPase activity by THs [44] triggers expulsion of cytoplasmic Ca^2+^, thus restricting the temporal window of Ca^2+^ uptake and hence the concentration of this ion within the cytoplasm. Calcium facilitates dephosphorylation of phosphorylated pyruvate dehydrogenase, thereby activating the enzyme [45]. Calcium also stimulates the entire oxidative phosphorylation cascade [46] within a specific range of ionic concentrations [47]. Therefore, it can be argued that the pro-metabolic non-genomic activities of THs which occur within seconds prime mitochondria for amplified activity of the electron transport chain. Another rapid, TH-driven, non-genomic phenomenon is stimulation of actin polymerisation [48,49,50]. While the exact mechanism of TH-induced F-actin formation remains largely unknown, it seems plausible that the supply of GTP to small GTPase organisers of actin polymerisation (e.g., Rho GTPases) could underpin this effect [51,52,53,54]. Given that GTP is produced in the citric acid cycle, F-actin formation could be potentially linked to Ca^2+^-mediated activation of pyruvate dehydrogenase with consequential activation of the downstream citric acid cycle. Further, THs amplify the production of ROS by mitochondria [36,37]. ROS-mediated oxidisation boosts the activity of small GTPases (in particular, Cdc42) by approximately three orders of magnitude by stimulating dissociation of GDP from inactive enzymes [55].

Complementing the cytoplasmic effects that energise citric acid cycle, THs directly activate the mitochondrial respiratory chain (Figure 1). Exposure of isolated mitochondria from hypothyroid animals to T3 stimulates oxidative phosphorylation within minutes [56,57]. Interestingly, this stimulatory effect is not confined to T3. The activity of the respiratory chain is rapidly enhanced upon exposure of mitochondria to T2, a degradation metabolite of T3 [58,59,60]. Activation of oxidative phosphorylation is, in part, due to binding of T2 to subunit Va of cytochrome c oxidase, which abolishes the allosteric inhibition of the complex by ATP [61]. An insight into the significance of disinhibition of cytochrome c oxidase by T2 is afforded by dissecting the molecular basis for allosteric inhibition of the complex by ATP. The capacity of the electron transport chain is mainly determined by the rate-limited activity of cytochrome c oxidase [62]. The complex operates in an excited or relaxed mode by integrating negative input from ATP [63]. The inhibition of cytochrome c oxidase by ATP is switched on by cAMP-dependent phosphorylation of the complex and switched off by Ca^2+^-activated dephosphorylation [64]. In a dephosphorylated excited state, the activity of the complex increases five- to tenfold [62]. In this excited mode, ROS generation by the electron transport chain occurs at a higher rate [63]. Likewise, a major outcome of T2-mediated disinhibition of cytochrome c oxidase is amplified generation of ROS by the mitochondrial electron transport chain [61]. Notably, the impacts of TH metabolites on mitochondrial metabolism are not mediated exclusively by T2. Decarboxylated metabolites of THs, thyronamines (T(0)AM and T(1)AM), at a concentration of >10^−7^ M amplify mitochondrial production of ROS by inhibiting complex III of the electron transport chain [65]. While the concentration of thyronamines in the developing brain is not known, a reported concentration of 10^−7^ M in the liver [66] provides assurance that the observed amplification of ROS by these compounds is a physiological effect. Following this line of reasoning, one may question the relevance of ROS to TH signalling.

It appears that critical nodes in eukaryotic signalling pathways have been populated by redox-sensitive proteins that abort downstream communication of signals [67]. Reprogramming of these proteins occurs by interaction with ROS, which transiently rewire the network topology of signalling pathways and facilitate downstream transmission of signals, as discussed by Vujovic et al. [67]. A focus on the role of ROS in facilitating the PI3K signalling pathway illustrates this point. Upon activation, PI3K catalyses the conversion of PIP2 to PIP3, which prompts Akt signalling [68]. Concurrent activation of PTEN by catalytic activity of protein phosphatase 2A [69] or by auto-dephosphorylation [70] antagonises the function of PI3K by converting PIP3 to PIP2. In an oxidising milieu (e.g., high [ROS]), PTEN becomes reversibly inactivated due to the formation of an intramolecular disulfide between the essential active Cys-124 residue and Cys-71 [71]. This transient inactivation of PTEN facilitates downstream communication of PI3K/Akt signals. The redox-mediated remodelling of signalling cascades enables control of the rate of biochemical events at a cellular level, thus giving rise to an adjustable cellular clock [67]. This insight could be utilised in revisiting the role of T2 binding to cytochrome c oxidase and relieving the ATP-mediated inhibition of the complex. Upon binding of T2, the transition of cytochrome c oxidase to an excited state induces a transient shift to an oxidising milieu owing to enhanced production of ROS [36,37]. While a shift to a pro-oxidising state will be short-lived as THs trigger production of antioxidants via the TR-mediated genomic pathway [34], recent findings suggest that transient amplification of ROS is sufficient to accelerate the rate of neuronal differentiation and that of brain development [18]. Hence, two parallel non-genomic arms of TH signalling, namely, cytoplasmic and mitochondrial effectors, combine to amplify production of ROS by mitochondria. These non-genomic effects are counterbalanced by a delayed adaptive genomic response invoked via TRs. The notion of biphasic activity of THs whereby TR-mediated effects antagonise the non-genomic impact of THs is bolstered by the finding that while hypothyroidism brings about a host of developmental anomalies, elimination of TRα1 prevents hypothyroidism-related anomalies in the developing cerebellum [14]. A plausible explanation for this observation is that elimination of TR-mediated antagonistic effects augments non-genomic effects of THs in a hypothyroid state, thus restoring normal developmental dynamics. The proposal that THs operate in a biphasic manner is further supported by dissection of the evolutionary interface of genomic and non-genomic functions of THs.

## 3. Thyroid Hormones: A Broad Evolutionary Perspective

The emergence of THs predates the evolution of the thyroid gland [72,73]. THs and associated metabolites are utilised by various species in the animal and plant kingdoms that lack a thyroid gland [72,74]. In the absence of an endogenous capacity to synthesise the entities, sea urchins utilise exogenous THs of plankton origin as an ecological cue to pace the tempo of development to availability of food [75]. In these animals, acquisition of exogenous THs accelerates metamorphosis [75]; the hormones are therefore considered to be ecological programmers of development [76]. Interestingly, the impacts of THs on larval development (i.e., inhibition of larval development and accelerated development of juvenile structures) are replicated by rearing larvae in a nutrient-rich condition [75]. The crosstalk between nutrient availability and TH production extends beyond the provided example of larval development. In mammals, the level of THs positively correlates with food availability, decreasing during periods of energy restriction and increasing upon access to energy substrates [77]. Another distinguishing feature of primitive TH signalling in invertebrates is the absence of a hormone receptor [73]. Therefore, it can be concluded that non-genomic effects of THs are more ancient that genomic effects that are mediated via receptors. The ancestral state of non-genomic effects compared to TR-mediated genomic effects is carried over to a functional level, where it manifests as dominance of non-genomic effects over genomic effects in driving development in the species with functional TRs. While initial reports suggested that the impact of THs on metamorphosis is mediated by TRs [78,79], subsequent gene knockout studies revealed that TRs are not essential for induction of metamorphosis by THs [78,79]. On the contrary, metamorphic transition is accelerated in the absence of TRs, a phenomenon that has been partially attributed to the removal of TR-mediated gene repression [78,79]. Evidence suggests not only that the evolutionary emergence and deployment of THs predates TRs [80] but also that developmental phenomena are predominantly regulated by more ancient TR-independent non-genomic activities of THs. The alternative interpretation that TRs were initially acquired and then lost in unicellular organisms and some basal metazoans suggests that non-genomic functions of THs are key to driving basic developmental events. Revisiting metamorphosis reveals another facet of primal TH signalling. It is noteworthy that mitochondrial dysfunction perturbs aspects of insect wing development during metamorphosis [81]. This effect is unlikely to be primarily related to an energy crisis for two reasons. It is known that the metabolic rate declines sharply at the beginning of metamorphosis and remains low until the completion of morphogenesis [82,83]. Further, consequences of perturbation of metamorphosis as a result of mitochondrial dysfunction are localised to the wings as opposed to a more generalised impact on metamorphosis, as is expected to occur in an energy crisis scenario [81]. A deeper insight into the role of mitochondria in TH signalling is provided by exploring the activity of the electron transport chain components in insect metamorphosis. Investigation revealed an unexpected finding that succinate rather than pyruvate is used as a metabolite of the respiratory chain [84]. In the same study, cytochrome c oxidase showed a higher turnover rate concurrent with initiation of metamorphosis [84]. Preferential utilisation of succinate by complex II of the electron transport chain is known to trigger a reverse electron flow to complex I, leading to significant production of ROS [85]. Likewise, impaired activity of cytochrome c oxidase (evidenced by a high turnover rate during metamorphosis [84]) contributes to reverse electron flow and ROS production [86]. Given the suggested role of ROS in driving metamorphosis [29,87,88], it seems plausible that succinate-mediated reversal of the electron transport chain and a high turnover of cytochrome c oxidase are deployed to increase ROS production and to tune the tempo of metamorphosis. By the same line of reasoning, contribution of THs to generation of ROS in metamorphosis [29] could be attributed to the impact of T2 on cytochrome c oxidase discussed in a previous section [63] or to a recently shown inhibitory impact of T3 and T4 on cytochrome c oxidase [89], which activates reverse electron flow [86].

Revisiting the conserved evolutionary functions of THs, it becomes apparent that THs in marine species function in the absence of TRs as an ecological currency for the availability of food. Accumulation of THs in these species boost the energetic capacity of mitochondria in anticipation of a nutrient-rich environment. Production of ROS by mitochondria, on the other hand, primes eukaryotic hosts for utilisation of the energy extracted from nutrients. As discussed, this occurs by ROS-mediated reprogramming of eukaryotic signalling cascades. It can be said that THs unlock a mitochondrial biochemical potential, and the resultant mitochondrial ROS then unlock the host’s biochemical potential. From this perspective, it can be envisaged that emergence of TRs was a subsequent evolutionary adaptation of metazoans that occurred in a stepwise manner to put an upper limit on the energetic capacity of a cell. This initially occurred by providing a delayed antagonistic input to counterbalance the non-genomic functions of THs on peripheral cells (e.g., by upregulation of antioxidants [34]). In the final stage of evolution and concurrent with emergence of a functional thyroid gland, the scope of activity of TRs expanded to provide a negative feedback input to dampen the endocrine synthesis of THs [90]. Considering the evolutionary history of THs and TRs, it is not surprising that abolishing TR-mediated genomic reprogramming boosts non-genomic effects of THs [38,39]. While the latter is an insight afforded by an experimental model, one may ask whether there is a biological mechanism to delay or transiently abolish TR-mediated transcriptional remodelling and to amplify non-genomic mitochondrial effects of THs.

## 4. Uncoupling of the Genomic and Non-Genomic Impacts of THs During Development

Nucleophagy is digestion of nuclear components in a manner that is similar to, but independent of, autophagy [91]. Nucleophagy is triggered within seconds [92] and facilitates reprogramming of nuclear function during differentiation [93] and trans-differentiation [92] by erasing aspects of the (epi)genomic memory of a cell. During nucleophagy occurring at an early stage of differentiation, the nucleus transiently resides in an uncoupled state from the cytoplasmic milieu, characterised by unresponsiveness to cytoplasmic cues. Notably, recent evidence suggests that nuclear uncoupling is triggered by mitochondria upon induction of neural differentiation [22]. To this end, the mitochondrial outer membrane transiently fuses with the nuclear membrane followed by acquisition and degradation of nuclear-encoded RNAs in the mitochondrial intermembrane space [22]. Additional consequences of the inter-organellar communication are transient inhibition of mitochondrial metabolic activity, suppression of ATP synthesis, and switching to ATP hydrolysis by F_1_F_0_ ATP synthase. Depletion of nuclear mRNAs and a reduced energetic budget for protein synthesis combined with enhanced autophagic flux bring about an effective nuclear uncoupling. Therefore, it can be argued that within a refractory window characterised by nuclear unresponsiveness during early differentiation, the TR-mediated genomic impact of THs will be attenuated, whereas mitochondrial activities will remain largely unaffected. During this refractory window, dominance of non-genomic effects reprograms the biochemical landscape of recipient cells by unbalanced overproduction of ROS. In this transient ROS^high^ state, the tempo of biochemical reactions is expected to increase, winding the “cellular clock” of development forward [67]. The discussion thus far concerns the impact of THs on individual cells. For THs to program brain development, the cell-level impacts of THs need to be translated to population-level dynamics. We therefore address how modulation of the behaviour of an individual cell could influence the collective behaviour of a population of cells during organisation of a developing brain. Self-organisation is the principle by which signals arising from the behaviour of individual cells are collected and integrated to shape the collective dynamics of a population of cells during organogenesis.

## 5. Cellular Self-Organisation and Brain Development: Tempo Informs Function and Spatial Organisation During Organogenesis

**Figure 2:**
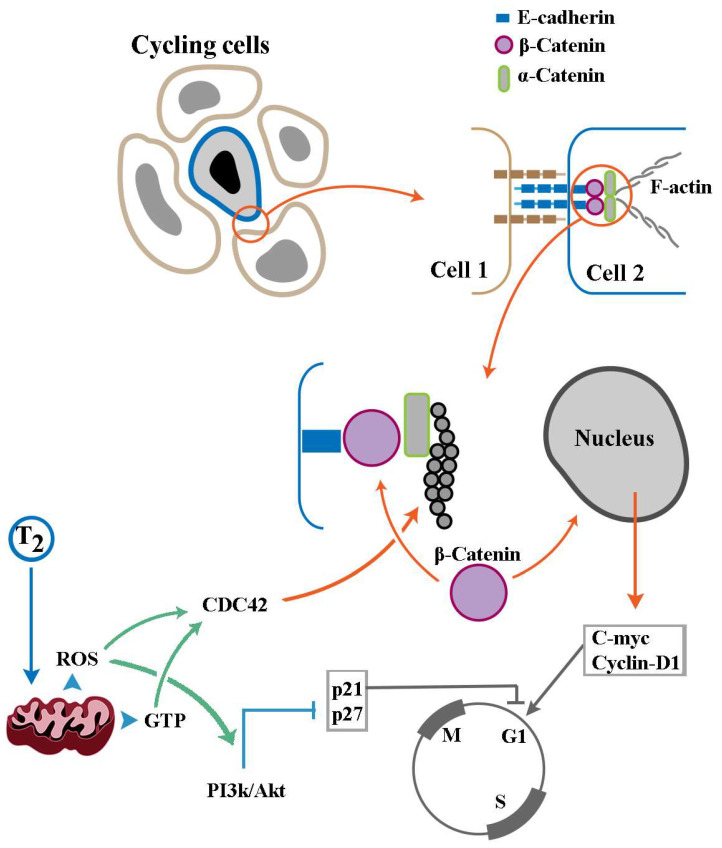
Mitochondrial regulation of cellular self-organisation dynamics. In a population of cycling cells, cadherin-based junctional complexes (top right) inform spatial organisation and the length of the G1 phase of cell cycle. Recruitment of β-catenin stabilises junctional complexes, while nuclear localisation of the protein trans-activates genes required for progression of the cell cycle. ROS also rewire and activate the PI3k/Akt pathway, a signalling cascade that represses the main inhibitors of the cell cycle. The mitochondrial supply of ROS and GTP activates small GTPases (e.g., Cdc42) to stabilise the association of F-actin with cadherin-based junctions. S, M, and G1 refer to the phases of the cell cycle (M: mitotic phase; S: DNA replication; G1: the gap between mitosis and DNA replication).

In the broadest sense, self-organisation refers to emergence of order at a global level through simple local interactions between components of a system [94]. In the context of organogenesis, recursive self-organising interactions between progenitor cells during organ development determine whether cycling cells remain in a proliferative pool or embark upon differentiation [95]. These interactions also determine where, along a migratory path within a developing embryo, cells assume a differentiated fate [96]. To this end, cadherin-mediated intercellular interactions appear to be central to spatial organisation as well as resolution of fate dichotomies and the resultant emergence of form and function by self-organisation [96,97]. To regulate spatial organisation, cadherin-based homo-polymeric interactions determine the directionality of collective cell migration by organising intracellular actin bundles [98] (Figure 2). Regulation of the cell cycle by cadherin-based junctions is underpinned by the dual functionality of β-catenin [99]. This protein not only serves as a structural component of cadherin-based junctions [99] but also trans-activates two major drivers of the G1 phase of the cell cycle, cyclin-D1 [100] and c-Myc [101] upon migration to the nucleus. Junctional storage of β-catenin generates a reserve protected pool of the protein, as the unbound free cytoplasmic protein is unstable and rapidly degraded by a destruction complex subsequent to phosphorylation by Gsk-3β [102]. Upon release from the junctional complexes, β-catenin faces two opposing fates. If Gsk-3β ks in a repressed state, β-catenin will migrate to the nucleus to trans-activate cyclin-D1 [100] and c-Myc [101]; otherwise, the protein will be phosphorylated and degraded, leading to a prolonged G1 phase of the cell cycle [103]. Therefore, factors that strengthen cadherin-based junctions (e.g., by promoting actin polymerisation [104]) and simultaneously inhibit Gsk-3β not only modulate spatial organisation of migrating cells but also reprogram the cell cycle properties (e.g., the length of the G1 phase) of these cells. One such factor is the small GTPase Cdc42 [105], which operates as a key organiser of actin polymerisation and an inhibitor of Gsk-3β [106]. A second pathway that regulates actin dynamics [107] and simultaneously contributes to stabilisation of free β-catenin is the PI3k/Akt signalling pathway [108,109]. To understand how non-genomic impacts of THs modulate self-organisation, it is necessary to address how the hormones regulate the dynamics of cadherin-based junctions by influencing the activity of key players such as PI3k/Ask and Cdc42.

We first focus on the capacity for junctional storage of β-catenin. Recruitment of β-catenin to cadherins is regulated at multiple levels [110]. In general, two antagonistic inputs regulate the stability of cadherin-based junctions. IQGAP1 dissociates α-catenin from the E-cadherin–β-catenin complex, destabilising it, whereas activated Cdc42 and Rac GTPases offset the effect of IQGAP1. A third player is calmodulin, which attenuates the binding of IQGAP1 to E-cadherin [111] in response to an increased intracellular concentration of Ca^2+^ [112], thus weakening E-cadherin junctional complexes [113]. Finally, enhanced actin polymerisation increases the stability of cadherin-based junctions [104]. THs interface with the described regulatory dynamics of cadherin-based junctions by stimulating mitochondrial production of ROS [36,37] (Figure 2). ROS-mediated oxidisation enhances the intrinsic rate of GDP dissociation from small GTPases, in particular Cdc42, amplifying the activity of the enzyme by approximately three orders of magnitude [55]. This impact of ROS is expected to stabilise cadherin-based junctions and enrich β-catenin at these junctions [18]. THs also stimulate actin polymerisation [48,49,50] by supplying GTP to small GTPase organisers of actin polymerisation (e.g., Rho GTPases) [51,52,53,54] along with ROS-mediated stimulation of GDP release to activate the enzymes [55]. By these activities, β-catenin will be enriched in cadherin-based junctions. TH-induced elevation of cytoplasmic [Ca^2+^] [41] could then associate with calmodulin to attenuate binding of IQGAP1 to E-cadherin [111] thus weakening E-cadherin junctional complexes [113] and triggering the release of β-catenin. The released β-catenin is expected to be stabilised by THs [114] via multiple mechanisms. First, mitochondrial ROS amplify the activity of Dishevelled (Dvl), a scaffolding protein that disrupts the GSK3β-mediated phosphorylation of β-catenin, leading to accumulation of the stabilised cytoplasmic protein [115]. The role of TH-amplified mitochondrial ROS in activating Cdc42 and PI3k/Akt, both of which stabilise β-catenin by regulating the activity of Gsk-3β [105,108,109], complements junctional enrichment of this protein, thus facilitating nuclear translocation of the protein to induce transcription of positive regulators of cell cycle [109]. However, positive input into the cell cycle is only effective when inhibitors of cell cycle dynamics are arrested. Accordingly, ROS-mediated rewiring of PI3k/Akt [67] by THs [116] prompts a series of phosphorylation events that inactivate inhibitors of cell cycle progression [117]. It is noteworthy that THs not only rewire the PI3k/Akt cascade but also activate the signalling pathway. Integrin αvβ3 has a T3-specific binding site that activates PI3k/Akt signalling pathway upon association with THs [118]. Once again, THs operate in a biphasic manner in regulating the cell cycle. Non-genomic mitochondrial effects of the hormones function as accelerators of the cell cycle [119], whereas TR-mediated transcriptional regulation induces cell cycle arrest [120]. This TR-mediated negative feedback is aligned with the antagonistic interaction between genomic and non-genomic consequences of TH signalling. In summary, mechanisms that regulate the tempo of biochemical events within an individual cell orchestrate self-organisation dynamics at a higher level by regulating the level and fate of β-catenin and the tempo of cell cycle. Following this line of reasoning, it is relevant to ask how accelerated cycling occurring as a consequence of the non-genomic impacts of TH signalling would alter the dynamics of self-organisation during brain development.

## 6. Heterochronic Signatures of THs in Brain Development

**Figure 3:**
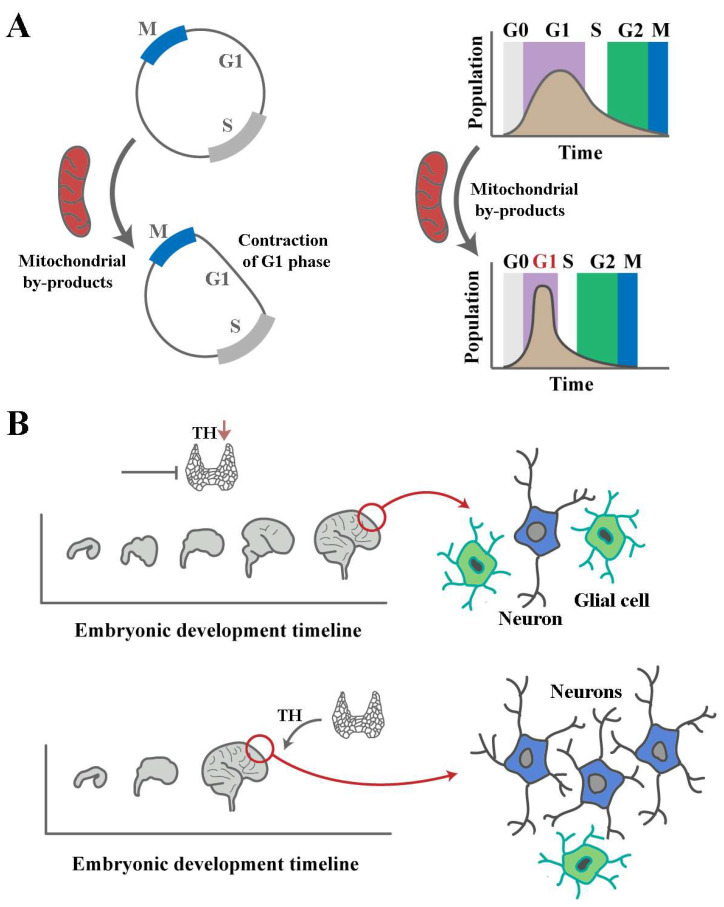
Heterochronic programming of brain development by THs. (**A**) Mitochondrial activity driven by THs accelerates progression through the G1 phase of the cell cycle [142] (**left**) by shortening this phase, winding developmental time forward at an individual cell level. At a population level, a shortened G1 phase leads to increased synchronicity of cycling cells, manifested as a reduced dispersion of the cell cycle state in a population of dividing cells in an arbitrary temporal window (**right**). In a synchronised cycling mode, proliferating cells are expected to be in a rather homogenous state with respect to the underlying mechanisms that propel the cell cycle. Accordingly, differentiation cues that are filtered via the lens of cell cycle dynamics will be interpreted in an analogous manner by proliferating cells, leading to adoption of similar differentiation fates by synchronised cells as opposed to a heterogeneity of differentiation outcomes in a non-synchronised cycling population. (**B**) The left schematic images show the development of the human brain in a hypothyroid state (**top**) as opposed to the achievement of neurodevelopmental milestones in the presence of normal levels of THs (**bottom**) in an arbitrary timeframe. A protracted development in hypothyroidism requires reprogramming of the cell cycle of neural progenitor cells. Given that the cell cycle regulates the differentiation tendency and spatial organisation of cycling cells, the need to reprogram the cell cycle in order to accelerate developmental time means that an altered tempo of organogenesis will be inevitably linked to a modified form and function of a developing brain. These pathological imprints of form and function in hypothyroidism could manifest as a reduced total number of differentiated cells or an altered differentiation outcome such as a change in the ratio of neurons to glial cells.

Heterochrony describes a reprogramming of ontogeny by changing the timing or the rate of developmental events [121,122]. From this perspective, THs can be classified as bistable heterochronic programmers of brain development. The bistability [123] of outcomes driven by THs stems from a competition between genomic and non-genomic functions of the hormones. Focusing on the impact of THs on the cell cycle clearly illustrates this notion. Accelerated progression through the G1 phase of the cell cycle leads to an enhanced proliferative capacity at an individual cell level and an amplified synchronicity of cycling neural progenitors at a population level [124] (Figure 3). The accelerated synchronised cycling tends to reduce the differentiation propensity of cells by two mechanisms. G1 phase dynamics are pro-differentiation, and rapid progression through this phase of cycle renders individual cells more resistant to differentiation cues [125,126]. At a population level, synchronised cycling restricts the differentiation-sensitive G1 phases of cycling cells to a narrow temporal window compared to the dispersion of G1 phases in an asynchronous population [124]. Hence, differentiation cues that arise during organogenesis are less likely to interact with synchronised rapidly cycling cells to trigger differentiation. The anti-differentiation non-genomic impacts of THs exerted via ROS are counterbalanced by TR-mediated genomic impacts of the hormone that induce cell cycle arrest [120]. Aside from modulation of cell cycle, there is some evidence that receptor-mediated activity of THs primes cycling cells for differentiation by upregulating the pro-neural transcription factor NeuroD in the developing cerebellum [127]. Owing to acceleration of cell cycle dynamics by THs [119], a deficiency of the hormone is expected to slow brain development (Figure 3). In an MRI-based study of the impact of thyroid disorders on brain development, hypothyroidism was found to be associated with reductions in bilateral total cerebellar and pallidum volumes [128]. In another study, both low and high maternal thyroid function were found to be associated with smaller total grey matter and cortical volumes in children [129]. One plausible explanation for the reduced size of the brain in hyperthyroidism is that an increased level of THs tips the balance of competition between genomic and non-genomic effects in favour of TR-mediated genomic effects, thus accelerating differentiation of neural progenitor cells and reducing the pool of proliferating cells. Given the absence of histological evidence in the latter study [129], the validity of this hypothesis remains to be assessed. In animal models, a reduction in the number of neurons is more evident in regions with significant neurogenic capacity, including the olfactory bulb and the granular layers of the hippocampus and cerebellum [130,131,132]. Corroborating the concept of heterochronic reprogramming in hypothyroidism (i.e., reduced tempo of development) is the finding that, in hypothyroidism, several developmental milestones of the brain are delayed. Reported phenotypes include disappearance of the subplate in the cortex [133], delayed regression of the external granular layer of the cerebellum [14], and delayed emergence of Cajal–Retzius cells in the cerebellum [134]. In the latter example, cerebellar development was surprisingly normal in mutant mice lacking TRα1 [14]. This observation is aligned with the suggestion that non-genomic effects of THs are responsible for programming brain development and that TR-mediated impacts counterbalance these non-genomic effects. However, an interpretation that the correction of brain development anomalies in hypothyroidism subsequent to removal of TRα1 is only an epiphenomenon and that the two events are not causally linked appears to be a valid competing scenario and further experimentation is needed to establish the causality of these observations. If such causality is confirmed, rigorous experiments are required to determine whether removal of TRα1 counterbalances the impacts of hypothyroidism by attenuating the negative feedback input on peripheral target cells or thyroid gland. Additional supportive evidence for bistable TH-mediated programming of ontogeny is afforded by the study of myelination during brain development. In agreement with the proposal for a bistable impact of THs on neurogenesis, while hypothyroidism causes delayed deposition of myelin [135,136,137,138], the inverse phenotype of accelerated myelination is observed in hyperthyroidism [139]. Therefore, it can be said that structural and functional modifications of a developing brain with hypothyroidism [140] are inevitable consequences of the altered tempo of development, which that requires heterochronic reprogramming of the cell cycle (Figure 3). A reprogrammed cell cycle not only alters the tempo of development but also modifies the differentiation outcome and spatial organisation of neural progenitor cells, some of which will persist as an irreversible signature of TH deficiency during brain development [141].

## 7. Conclusions and Future Directions

Here, we provide evidence for a proposal that THs orchestrate the development of the brain by regulating the tempo of cellular self-organisation. The basic tenets of the proposal are as follows:

1. THs orchestrate brain development by controlling the balance of competition between receptor-mediated genomic and non-genomic mitochondrial effects.
2. A transient suppression of nuclear dynamics facilitates a predominance of non-genomic impacts of THs over TR-mediated genomic effects.
3. To assume a morphogenic role, THs reprogram mitochondria to produce ROS at an amplified rate.
4. The transient shift to an oxidising milieu as a result of TH signalling leads to rewiring of certain signalling pathways, an accelerated cell cycle, and enhanced tempo of cellular self-organisation.
5. An enhanced tempo of self-organisation in TH signalling is a major determinant of the emergence of spatial and functional signatures of cellular self-organisation.
6. In hypothyroidism, the reduced tempo of cellular self-organisation underpins key anatomical and functional alterations of a developing brain.

We suggest that TH signalling during development activates two antagonistic outcomes, namely, direct mitochondrial reprogramming and an adaptive remodelling of transcriptional profile to offset the mitochondrial activities. It is the balance of competition between these two outcomes that determines the tempo of self-organisation and emergence of form and function in a developing brain.

A key step in validating the presented hypothesis is to dissect the interface of non-genomic and genomic functions of thyroid hormones at the level of peripheral cells using rigorous experimental designs. Preliminary analyses could be performed in eukaryotic cells devoid of mitochondrial DNA (rho0 cells) [143,144]. Exposure of rho0 cells to different metabolites of THs could provide valuable information regarding the role of mitochondria in mediating the impacts of THs. A key confounding factor in these experiments is expected to be the crosstalk between mitochondria and the nucleus. For example, Notch1 operates as a thermal sensor of mitochondrial activity that regulates gene expression in a temperature-dependent manner [23]. Therefore, investigation of the role of THs in rho0 cells should be confined to short temporal windows to rule out the possibility of such indirect confounding effects. Advancing the experiments to an organism level, however, will be more challenging. In these experiments, reprogramming the electron transport chain via genetic tools [145] could provide useful insights regarding the role of mitochondria in mediating the impacts of hypothyroidism on brain development.

## Data Availability

Data is contained within the article.

## Abbreviations

TH	Thyroid hormone
TR	Thyroid hormone receptor
ROS	Reactive oxygen species
CoR	Co-repressor complex
CoA	Co-activator complex
PTEN	Phosphatase and tensin homolog
PI3k	Phosphoinositide 3-kinase
Akt	Protein kinase B
PIP2	Phosphatidylinositol 4,5-bisphosphate
PIP3	Phosphatidylinositol (3,4,5)-trisphosphate
Dvl	Dishevelled
IQGAP1	Ras GTPase-activating-like protein IQGAP1
